# Morphology captures diet and locomotor types in rodents

**DOI:** 10.1098/rsos.160957

**Published:** 2017-01-18

**Authors:** Luis D. Verde Arregoitia, Diana O. Fisher, Manuel Schweizer

**Affiliations:** 1 Naturhistorisches Museum Bern, Bernastrasse 15, Bern 3005, Switzerland; 2 School of Biological Sciences, University of Queensland, St Lucia, Queensland 4072, Australia

**Keywords:** discriminant analysis, ecomorphology, non-metric multi-dimensional scaling, phylomorphospace, size correction

## Abstract

To understand the functional meaning of morphological features, we need to relate what we know about morphology and ecology in a meaningful, quantitative framework. Closely related species usually share more phenotypic features than distant ones, but close relatives do not necessarily have the same ecologies. Rodents are the most diverse group of living mammals, with impressive ecomorphological diversification. We used museum collections and ecological literature to gather data on morphology, diet and locomotion for 208 species of rodents from different bioregions to investigate how morphological similarity and phylogenetic relatedness are associated with ecology. After considering differences in body size and shared evolutionary history, we find that unrelated species with similar ecologies can be characterized by a well-defined suite of morphological features. Our results validate the hypothesized ecological relevance of the chosen traits. These cranial, dental and external (e.g. ears) characters predicted diet and locomotion and showed consistent differences among species with different feeding and substrate use strategies. We conclude that when ecological characters do not show strong phylogenetic patterns, we cannot simply assume that close relatives are ecologically similar. Museum specimens are valuable records of species' phenotypes and with the characters proposed here, morphology can reflect functional similarity, an important component of community ecology and macroevolution.

## 1. Background

Understanding how morphological features vary among species with different ecological habits is not trivial, given our limited understanding of the ecology of many living species, even in well-studied groups such as mammals. In vertebrates, variation in ecological attributes such as feeding and substrate use is commonly associated with variation in morphology [1]. The form–function relationship also has elements of phylogenetic relatedness, chance and common adaptive response that remain understudied [2,3]. With the increasing detail and availability of phylogenetic data, the relationship between form and function can be studied in an evolutionary context [4].

From an adaptive evolutionary standpoint, ecology and morphology are linked by common functional demands [5]. In some cases, however, morphology may simply reflect retained ancestral features rather than adaptation to present conditions [6]. Because evolution is a branching process and traits tend to be more conserved than random [7], phylogenetic relatedness is assumed to reflect ecological similarity (i.e. phylogenetic signal). The assumption of strong phylogenetic signal in ecological traits has led to studies that use ecological, morphological and phylogenetic metrics interchangeably. For example: when morphology is used as a surrogate for species' functional roles in ecological assemblages [8], and phylogenetic distance between species as a measure of ecological similarity [9]. However, species do not necessarily retain ancestral ecological characteristics [10,11].

Phylogeny, morphology and ecology are interconnected—a squirrel is still a squirrel in each of these regards, so it is not a matter of one approach being better than another, but a question of examining the phylogenetic patterns in morphology and ecology in order to make meaningful ecological or evolutionary interpretations. Instead of using only morphology or phylogenetic affinities to infer ecological, and thus functional similarity between species, we need to first study the functional relationships between morphology and ecology in a phylogenetic comparative context. Once we identify ecologically relevant morphological variables, we can interpret similarity between species in ecological terms and use this measure of similarity to address other ecological questions (e.g. competition, disparity and biological invasions). We can measure large numbers of morphological traits using natural history collections and can interpret them in functional terms, even for ecologically undescribed and poorly known species.

Rodents are the most diverse extant mammal group, with over 2200 described species [12]. The order spans a wide array of body sizes and shows great diversity in locomotor habits and feeding ecology, having evolved aquatic, arboreal, fossorial, jumping and gliding forms, with a wide array of feeding preferences that include animal- and plant-eating specialists. For small mammals, relatively subtle changes to the morphology of the bones and soft tissues can have dramatic functional consequences, making rodents a good example of ecological specialization with and without radical morphological changes [13]. The average rodent can climb, dig and swim without extensive morphological specializations [14]. Nonetheless, specialist forms have evolved (often independently) and can be found in nearly every non-marine habitat. Rodents are also major ecosystem components, given their position in food chains and their importance in soil tillage, seed dispersal and pollination [15].

Feeding and substrate use (diet and locomotion) are important ecological attributes which have been related to morphology in rodents [16,17]. In most cases, however, the sample of species analysed was low or constrained to a single family, using characters that are not accessible without specialized equipment or postcranial skeletal material that is often not available at natural history collections, despite the call for skin-plus-skeleton preparation as the standard mammalian museum specimen [18]. Previous studies on rodents have identified consistent differences in morphology that relate to functionally important traits, and found differences in postcranial skeletal and appendicular characters among climbing, digging, swimming and jumping species [13,14,19]. The versatility of rodent feeding behaviours is also evident in their feeding apparatus [20]. Molar crown descriptors discriminated the diets of extant muroids [21] and incisor morphology reflected the diet of 11 caviomorph genera [22]. A morphometric analysis related dental morphology to diet, using only the outlines of the first upper molar of extant murines [23].

In this study, we examine the morphological variation in rodents with different feeding and locomotor strategies. We address the existing gaps in establishing functional relationships between species' morphology and ecology in a quantitative, phylogenetic framework. We aim to: (i) examine and interpret the relationship between morphology and ecology in rodents, using feeding and locomotion strategies, while taking phylogenetic relationships into account; (ii) validate the ecological relevance of an accessible set of morphological traits with a sample that includes all ecomorphs and multiple families; (iii) identify how these traits vary between groups and (iv) infer the ecology of ecologically uncharacterized species using morphological data. If our chosen characters are ecologically relevant and subject to strong selective pressures, we should not find strong phylogenetic patterns. Ecologically and morphologically similar species in this order should converge in morphospace when we account for the phylogenetic component.

## 2. Material and methods

### 2.1. Morphological data

**Figure 1.**
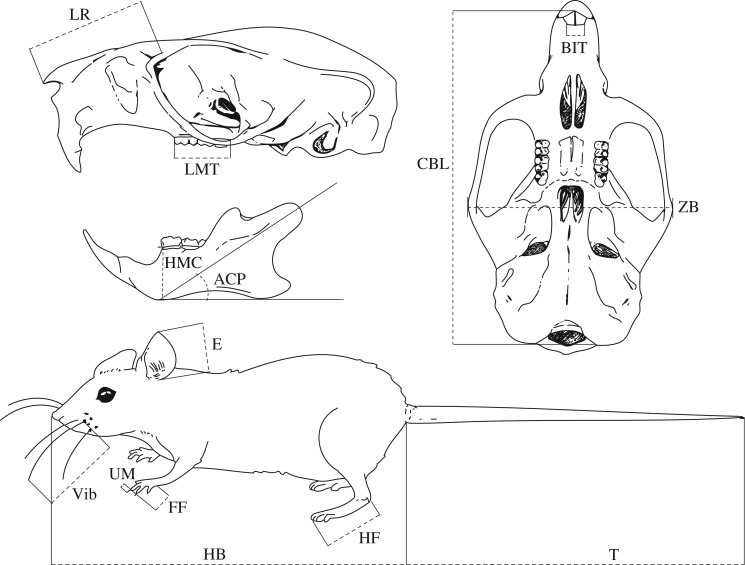
Morphological characters examined. See table 1 for descriptions.

**Figure 2.**
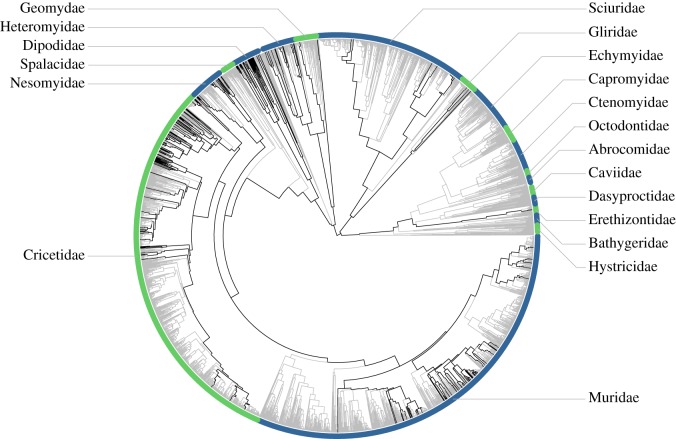
Sampled species in this study (black lines) within the phylogeny of Rodentia (one randomly selected tree from a set of 100), derived from the species-level mammalian phylogeny of Faurby & Svenning [24]. Families with 10 or more species are marked with coloured bands and labelled.

We collected data on seven craniodental and seven external measurements (figure 1 and table 1) for 208 rodent species from 10 different families (figure 2; electronic supplementary material, table S1), using data from previous studies provided by A. Miljutin, and from a total of 2014 specimens examined by LDVA at natural history collections (electronic supplementary material, appendix­). These 14 characters were identified to be ecology-dependent in a previous study that considered 48 characters (25 craniodental and 23 external) and their correlations with well-defined feeding and locomotor and ecological strategies for the rodent fauna of the Baltic region [25]. In the same study, the chosen traits also showed high interspecific variability and low intraspecific variability. Our choice of species was driven by the availability of undamaged skins and skulls at the collections, but we consider that these species are representative of the overall taxonomic and ecological diversity within the rodent fauna of Australia and neighbouring islands (Sahul), the Baltic region, Madagascar and Mesoamerica.

**Table 1.** Description of morphological characters. All characters were measured in millimetres except ACP, measured in degrees.

	external characters
HB	*head and body length*: distance from the tip of the nose to the base of the tail
T	*length of tail*: distance from the base of the tail to its tip without terminal hairs
E	*ear length*: distance from the basal notch to the tip without terminal hairs
Vib	*length of vibrissae*: length of the longest mystacial vibrissa from the base to the tip in its natural position
HF	*length of hind foot*: distance from the heel to the tip of the longest digit without claw
FF	*length of forefoot*: distance from the notch between the radius and carpus to the tip of the longest digit without claw, measured parallel to the manual axis
UM	*length of forefoot claw*: distance from the base of the longest claw on its inferior surface to the tip

	craniodental characters
CBL	*condylobasal length*: distance from the border between the anterior surface of the upper incisors and intermaxilla to the posterior surfaces of the occipital condyles measured parallel to the cranial axis
LR	*length of rostrum*: distance from the tip of the nasal bones to the anterior edge of the zygomatic arch, measured level with the nasals and parallel to the cranial axis
ZB	*zygomatic breadth*: greatest breadth across the zygomatic arches
BIT	*breadth across incisor tips:* distance across the tips of the incisors
LMT	*alveolar length of maxillary tooth row*: distance from the anterior edge of the alveolus of the maxillary tooth row's first tooth to the posterior edge of the alveolus of the third molar
HMC	*height of mandibular corpus*: distance from the anteriodorsal part of the first molar to the ventral surface of the mandibular corpus, measured perpendicular to the masticatory surface of the mandibular tooth row
ACP	*angle of condylar process*: angle between the tangent to the ventral surface of the mandibular corpus parallel to the masticatory surface of the mandibular tooth row and the line connecting the tangent's contact point with the axis of the mandibular condyle

We recorded standard external measurements (body mass, head and body length, tail length, ear length and length of the hind foot; table 1) from specimen labels. All other linear measurements were taken by LDVA to the nearest 0.01 mm using digital callipers (Fowler UltraCal Mark IV). The angle of the condylar process (ACP) was measured from digital photographs of the lateral view of the mandible using ImageJ (National Institutes of Health, Bethesda, MD, USA).

### 2.2. Phylogenetic trees

To quantify the inferred evolutionary relationships between the species that we examined, we extracted 100 trees (at random) from a set of 1000 generated under a heuristic–hierarchical Bayesian framework in a recent species-level mammal phylogeny [24]. In this approach, species with large quantities of molecular data are placed in the phylogeny according to these data, while species with lower quantities of data are added under steadily stricter restrictions depending on what is known about their affinities. Each tree represents a slightly different phylogenetic hypothesis, so covariance among species across tree replicates may affect any comparative analyses. Consequently, rather than using a single tree and assuming that the relationships are known without error, we repeated all our comparative analyses across multiple trees. This approach allows us to examine the variation in parameter estimates arising from differences among tree topologies [26].

### 2.3. Ecology data

Using published data, we classified each species into categories that reflect their substrate use and feeding strategies. We used information on feeding habits, primary dietary items, and use of different locomotor modes and substrate types to assign species into four dietary categories and seven locomotor groups (tables 2 and 3), using a modification of existing categories [14,20,27]. We did not consider omnivory as a diet type, and instead assigned most species traditionally described as omnivorous into the generalized herbivore (GH) category, recognizing that generalized herbivores opportunistically consume animal matter and fungi. We were able to assign 185 species into a locomotor mode using at least one source. For feeding strategies, detailed natural history information is sparser and we could only assign 166 species into a diet class (electronic supplementary material, appendix). Species for which we could not confidently assign ecological categories were classified as unknown (U), and their diet and locomotion were predicted using morphology with the predictive framework that is built into discriminant analyses.

**Table 2.** Locomotor categories.

terrestrial (T)	Rarely swims or climbs, may dig to make a burrow (but not extensively), may show saltatory behaviour (quadrupedal only), never glides (e.g. rats and mice)
semiaquatic (Sa)	Regularly swims for dispersal, escape or foraging (e.g. beavers and muskrats)
arboreal (A)	Capable of and regularly seen climbing for escape, shelter or foraging (includes scansorial species; e.g. tree squirrels and erethizontid porcupines)
semifossorial (Sf)	Regularly digs to build burrows for shelter, but does not forage underground (e.g. ground squirrels)
fossorial (F)	Regularly digs to build extensive burrows as shelter or for foraging underground (e.g. gophers and mole rats). Displays a predominantly subterranean existence
ricochetal (R)	Capable of jumping behaviour characterized by simultaneous use of the hind limbs, commonly bipedal (e.g. kangaroo rats)
gliding (G)	Capable of gliding through the use of a patagium, commonly forages in and rarely leaves trees (e.g. flying squirrels)

**Table 3.** Diet categories.

carnivore (C)	Diet composed primarily of animal matter, including some vertebrate or larger invertebrate material (e.g. grasshopper mice)
insectivore (I)	Diet composed of animal matter, but primarily small arthropods (insects and chelicerates), grubs or earthworms (e.g. shrew mice)
generalized herbivore (GH)	Diet composed primarily of plant matter, mostly soft leafy vegetation, fruits or seeds. Diet also includes fungi and animal matter in varying amounts (e.g. spiny pocket mice)
specialized herbivore (SH)	Diet composed exclusively of plant matter, including large amounts of particularly fibrous or difficult to process plants (e.g. grass, bark or roots) or dust and grit (e.g. Australian broad-toothed rat)

### 2.4. Data processing and analysis

We carried out all statistical tests in R v. 3.2.5 [28]. All data and code are provided as the electronic supplementary material (see Data accessibility section). We used the package *dplyr* [29] for all data processing and manipulation workflows, and we mention the relevant functions and packages for all our statistical analyses below.

### 2.5. Size correction

The species in our sample ranged in size (body mass) from 10 (pygmy mice and jerboas) to over 10 000 g (beaver), a 2000-fold range covering the range of sizes of most extant rodents [30]. To remove the variation in trait values that occurs simply because species vary in size, we chose to analyse the residual variation in each character after removing the correlation with a measure of overall body size. Out of three highly correlated (non-central correlations greater than 0.95) candidate proxies of body size (body mass, condylobasal length and head–body length), we selected body mass as a measure of overall body dimension because we considered it a suitable whole-body descriptor that has known relationships with species' ecology. With each of the 100 trees in our set, we performed phylogenetic generalized least-squares (PGLS) regressions [31,32] of the log-transformed morphological traits (except the angle of the condylar process, which we did not expect to vary with size) and body mass, using the ‘lambda’ method [33] to obtain the error structure for the models with the *phyl.resid* function in the *phytools* [34] package. We averaged the set of residuals from each of the 100 trees to incorporate phylogenetic uncertainty into the size-correction process [35].

Using residuals from a regression to deal with size variation removes size and also allometric variation from the dataset, leaving ‘non-allometric shape’ information. This focus on non-allometric morphological variation in relation to ecology may limit our interpretation, because an animal's size is likely to influence the niches it can occupy. We used the *phylANOVA* function in *phytools* [34] to run simulation-based phylogenetic analysis of variance [36] to test for differences in body size (using two proxies: body mass and condylobasal length) among species with different diets and locomotion modes. If ecological habits relate strongly with body size, we would need to use an alternative size-only correction that keeps allometric variation such as the log-shape ratio approach of Mosimann [37] to examine how much diversity and homoplasy there is that has evolved other than by simple allometric scaling during speciation.

### 2.6. Ecology and morphology

Before using discriminant analyses to examine the morphological changes associated with differences in diet and locomotion, we used phylogenetic multivariate analyses of variance across the set of trees (phylogenetic MANOVAs) to first test whether or not we had significantly different ecological groups. To fit the models, we used a Procrustes distance PGLS (*procD.pgls* function in the *geomorph* [38] package) on the size-corrected data. This test is designed to take a matrix of morphological variables as a response while including the phylogeny in the error term.

Afterwards, we were interested in examining the between-class variance in the data, so we used discriminant analyses. Discriminant analyses are supervised methods, using known class labels to maximize the separation among species belonging to different ecological classes. We conducted a phylogenetically informed, flexible discriminant function analysis (pFDA) to examine the relationship between morphological variables and ecological categories. Flexible discriminant analysis is a generalization of linear discriminant analysis; it reduces a discrimination problem to a regression problem [39], making it compatible with a PGLS framework [40,41]. pFDA has been used to relate ecology with morphology for different taxonomic groups [42–46]. Standard and phylogenetic discriminant analyses rely on functions from the R package *mda* [47].

The degree of phylogenetic bias removed (assuming Brownian motion evolution) in pFDA is determined by Pagel's lambda. The optimal value of lambda for a given tree topology can be found by identifying the value that minimizes the residual sum of squares of the linear fit between the phylogenetically corrected matrices containing the continuous and categorical data for each species [42]. For each of the 100 trees, we estimated the optimal value of Pagel's lambda. A lambda value of zero indicates that phylogeny has no importance in the model, equivalent to a non-phylogenetic analysis. If lambda equals one, phylogeny is an important component of the model, with the residuals following a Brownian motion model of evolution [43,48]. In this case, phylogenetic correction is necessary to ensure that the resulting projections into discriminant space are evolutionarily orthogonal. Previous studies that used pFDA showed that even small differences in the lambda values used to account for phylogenetic covariance among species led to different sets of classifications and error rates [49], so despite the low lambda values (see Results), we repeated all analyses using both phylogenetic and standard (non-phylogenetic) flexible discriminant analysis.

After running the discriminant analyses, we used the R package *DiscriMiner* [50] to calculate Wilks' lambda and evaluate the discriminant power of the independent variables. Wilks' lambda represents the proportion of the total variance in the discriminant scores not explained by differences among groups for a given variable. Wilks' lambda statistic can be mathematically adjusted to a statistic which has approximately an *F* distribution for significance testing. Variables with low values for Wilks' lambda have higher discriminatory power.

### 2.7. Morphospace

We measured an angle in addition to the linear measurements, and because there is no natural relation among the scales and units of measurement for these variables, the morphospace resulting from our flexible discriminant analysis approach would not have a Euclidean structure [51]. This means that a meaningful interpretation of the spread and spacing of taxa within this morphospace is not guaranteed (for example: interpreting the distances between points on a two-dimensional plot as morphological dissimilarity). Discriminant function analyses and analyses of variance with mixed angular and linear data are statistically valid, and they provide important and useful information about how morphology varies among different ecologies, the likely ecology of taxa according to their morphology, and the predictive power of different measurements. However, reducing the dimensionality of morphological data for visualization is a common and helpful tool in ecomorphology. We complement our phylogenetic discriminant function approach with a non-metric multi-dimensional scaling (nMDS) analysis to visualize the variation in morphological variables as described by two nMDS axes, in which ‘closer’ morphologies are more similar than more ‘distant’ morphologies [51]. We used Sammon's nonlinear mapping [52,53] with the *sammon* function in the *MASS* package [52] to collapse multi-dimensional morphological data onto a two-dimensional space while minimizing a stress function that reflects goodness of fit. Because nMDS is an unsupervised analysis, we labelled the species in the resulting morphospace with the ecological categories predicted by the discriminant analyses.

### 2.8. Phylogenetic signal

Quantifying the phylogenetic patterns in all of our data is an important first step that can help us interpret all the results in this study. In addition to estimating the optimal value of lambda for pFDA, we measured phylogenetic signal separately for body size, morphological measurements and ecological habits. For body size, we calculated the *K* statistic of Blomberg *et al.* [11] with our body mass data. For morphological data, we calculated *K*_mult_ [54], a generalization of the *K* statistic for multivariate data. *K* and *K*_mult_ are standardized metrics that quantify the degree to which variation in a trait or set of traits is explained by the structure of a given phylogeny. *K* < 1 indicates that closely related species resemble each other less than expected under a Brownian motion model of trait evolution. *K *> 1 means that closely related species are more similar than predicted by the model. To quantify the phylogenetic signal for the discrete dietary and locomotor categories, we followed a recent proposal [55] to incorporate evolutionary models into Mantel tests in order to resolve previous issues of lower statistical power and higher type I error rates.

## 3. Results

### 3.1. Size correction

The PGLS models had low variation in the slope and intercept estimates across the 100 tree replicates, suggesting that our size corrections were robust to phylogenetic uncertainty (electronic supplementary material, table S2). We used the means of the residuals across all trees so that our conclusions did not depend on the assumption of any single tree being ‘correct’. We found no differences in body size among species with different diet types (phylogenetic ANOVA; *p* > 0.05 for 81/100 trees for body mass and *p* > 0.05 for 99/100 trees for condylobasal length), and body size did not differ among locomotor modes either (phylogenetic ANOVA; *p* > 0.05 for 100/100 trees for body mass and *p* > 0.05 for 100/100 trees for condylobasal length). In the light of these results, we consider the use of residuals for further analyses to be appropriate.

### 3.2. Phylogenetic signal and phylogenetic discriminant analyses

We found weak (less than expected under a Brownian motion model) but non-random phylogenetic signal for body size (mass in grams), craniodental measurements and external measurements (table 4). Using the evolutionary-model Mantel test [55], we found no evidence of phylogenetic signal in diet type (very weak, non-significant Mantel correlations between phylogenetic and trait distances). Locomotor modes appear to be more conserved, and we found evidence of low phylogenetic signal for these habits (weak but significant Mantel correlation; table 4).

**Table 4.** Phylogenetic signal. Summary statistics for various measurements of phylogenetic signal run across a set of 100 phylogenetic trees.

character(s)	median value	minimum value	maximum value	metric	significance testing^a^
optimal lambda (diet)	0.04	0.04	0.07	Pagel's lambda	n.a.
optimal lambda (locomotion)	0.04	0.03	0.05	Pagel's lambda	n.a.
body size (mass in grams)	0.18	0.01	0.62	Blomberg's *K*	98/100
craniodental and mandibular measurements	0.18	0.01	0.65	*K*_mult_	98/100
external measurements	0.20	0.02	0.65	*K*_mult_	99/100
diet type	−0.10	−0.11	−0.08	EM Mantel test (Mantel correlation)	0/100
locomotor mode	0.36	0.35	0.38	EM Mantel test (Mantel correlation)	100/100

^a^The number of trees for which tests against a null hypothesis of no phylogenetic signal (e.g. by permutation tests) attained statistical significance (*α* = 0.05).

Optimal lambda values for diet type and locomotor mode were close to 0 across the tree set (table 4). Considering the predicted classes and the class means in discriminant space, the classification ability and results of FDA were quantitatively indistinguishable from pFDA even across 100 slightly different phylogenetic hypotheses. The discriminant scores for standard and phylogenetic FDA showed high correlation (always above 0.95; Pearson's correlation), meaning no discernible shift between the position of species in (affine) morphospace and phylomorphospace.

### 3.3. Diet

Running phylogenetic MANOVAs, we found that morphology differs significantly among diet types (d.f. = 3, *p* < 0.05 for 98/100 trees). Using standard flexible discriminant analysis for dietary categories, morphological data allowed for the correct classification of 89% of species into their diet categories (cross-validated error rate from ten random partitions of the training data). Discrimination was best for generalized herbivores and specialized herbivores (GH and SH; 99 and 81% correct classification, respectively), and worst for carnivorous and insectivorous species (C and I; 50% correct classification). The first discriminant function (DF1) accounted for 76.38% of the total between-group variance. DF2 accounted for 16.98% of the between-group variance. DF3 explained the remaining 6.64% of the within-group variance.

Examining the correlations between the morphological characters and the linear discriminants, measurements related to the masticatory apparatus (ACP, LMT, BIT and HMC) had the highest influence on the discriminant axes (table 5). Several variables load onto the different DFs simultaneously, but DF1 was driven mainly by ACP. Along this axis, specialized herbivores (e.g. voles) with very pronounced condylar processes, longer maxillary tooth rows, wider incisors and a thicker mandible had low scores, while animal-eating (insectivorous and carnivorous) species with the least pronounced condylar processes and more gracile mandibles scored highest on DF1. DF2 mostly reflected the length of the maxillary tooth row. This axis captured the distinctive combination of features of the New Guinea moss mice *Pseudohydromys ellermani*, which have a short, stout rostrum with retracted nasals and extreme reductions in molar size and number [56].

**Table 5.** Discriminant structure. Correlations between morphological variables and discriminant functions for the standard (non-phylogenetic) FDA on diet type and locomotion mode. Figures in italics represent significant correlations (Holm-corrected *p*-values smaller than 0.05).

	diet			locomotion					
character	DF1	DF2	DF3	DF1	DF2	DF3	DF4	DF5	DF6
LR	*0.28*	0.2	*0*.*29*	−*0*.*48*	0.14	*0*.*33*	−0.06	*0*.*5*	−0.01
ZB	−0.14	*0*.*32*	−0.11	*0*.*41*	−0.03	−*0*.*58*	−*0*.*35*	0.05	−*0*.*39*
BIT	−*0*.*52*	−*0*.*36*	−0.23	−*0*.*36*	−*0*.*25*	−0.09	−*0*.*28*	*0*.*25*	0.03
LMT	−*0*.*64*	*0*.*4*	*0*.*34*	−0.08	0.09	0.06	0.11	0.13	−*0*.*54*
HMC	−*0*.*48*	*0*.*43*	−*0*.*46*	0.15	−0.08	−*0*.*32*	−*0*.*3*	−0.19	0.17
ACP	−*0*.*88*	0	−0.19	0.03	−*0*.*47*	−0.24	*0*.*27*	−0.05	0.2
T	*0*.*45*	0.12	0.04	0.24	*0*.*5*	−*0*.*35*	*0*.*45*	*0*.*44*	−0.15
E	*0*.*42*	*0.4*	0.06	*0*.*38*	*0*.*6*	0.21	0.03	0.2	−0.06
Vib	*0*.*49*	*0*.*3*	0.07	*0*.*52*	*0*.*57*	−0.03	−0.03	−0.06	−0.03
HF	*0*.*32*	0.11	0.15	*0*.*78*	0.19	−0.23	0.24	0.21	−0.18
FF	−0.06	−0.19	0.19	−*0*.*63*	0.12	−*0*.*36*	−0.02	−0.06	−0.04
UM	−*0*.*25*	−0.09	−0.13	*0*.*46*	−*0*.*3*	−*0*.*43*	−*0*.*37*	0.05	0.1

When we quantified the discriminant power of the independent variables, most variables contributed to the discrimination of species into dietary classes to some extent. All the variables except forefoot length were significantly correlated with at least one of the discriminant axes. All the measurements except forefoot length (FF) showed significant contribution to the discriminant functions, and the two variables with the highest power were ACP and LMT (table 6).

**Table 6.** Discriminant power of independent variables.

	diet			locomotion		
character	Wilks' lambda	*F*-statistic	*p*-value	Wilks' lambda	*F*-statistic	*p*-value
ACP	0.426	72.721	0	0.819	6.579	0
LMT	0.611	34.413	0	0.966	1.044	0.398
HMC	0.715	21.472	0	0.899	3.368	0.004
BIT	0.738	19.144	0	0.763	9.288	0
Vib	0.788	14.498	0	0.56	23.397	0
E	0.807	12.879	0	0.618	18.455	0
T	0.847	9.728	0	0.67	14.713	0
LR	0.911	5.261	0.002	0.695	13.082	0
HF	0.914	5.05	0.002	0.411	42.779	0
ZB	0.945	3.167	0.026	0.657	15.551	0
UM	0.947	3.026	0.031	0.624	17.972	0
FF	0.976	1.325	0.268	0.604	19.572	0

### 3.4. Locomotion

With the phylogenetic MANOVAs, we found that morphology tends to differ among locomotion modes (d.f. = 6, *p* < 0.05 for 77/100 trees), but this result was somewhat sensitive to uncertainty in the phylogenetic hypotheses. Morphological data allowed for the correct classification of 69% of species into their ‘correct’ locomotor modes by the FDA (cross-validated error rate). All fossorial, gliding and ricochetal species were correctly classified, followed by terrestrial species (T; 85% correct classification), semiaquatic species (Sa; 83%), semifossorial species (Sf; 70%) and finally arboreal species (A; 66%). The first discriminant function (DF1) accounted for 71.95% of the total between-group variance. DF2 accounted for 14.27% of the between-group variance. External characters, and appendicular measurements in particular, had the strongest correlations with the linear discriminants. DF1 separated jumping species with elongated hind feet (HF), longer claws on the forefeet (UM) and shorter forefeet (FF) than those with other locomotion modes. DF2 had strong correlations with ear (E), tail (T) and vibrissae length (Vib). Arboreal species with long tails, ears and vibrissae score higher on this axis, while semifossorial, semiaquatic and fossorial species score lower (table 5). All the variables except LMT had a significant contribution to the discriminant functions, and the variables with the highest discriminant power based on Wilks' lambda were HF, FF and Vib (table 6).

### 3.5. Morphospace

**Figure 3.**
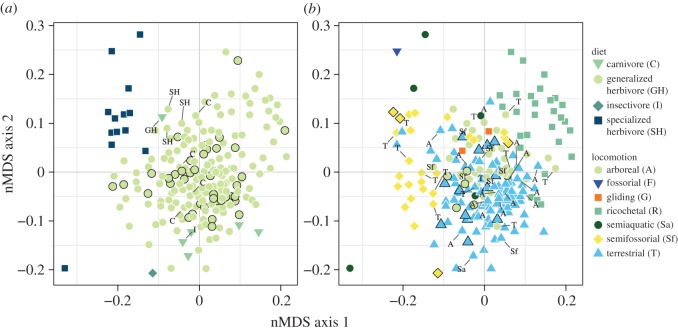
Morphospace plot of nMDS ordination (Sammon's nonlinear mapping) of morphological data for 208 species of rodents based on 12 variables. Each species is labelled (colour and shape) with its predicted diet (*a*) and locomotion (*b*) from a flexible discriminant analysis (FDA). Species with no information on ecological habits in the training dataset (42 for diet and 35 for locomotion) are indicated by black outlines. Differences in classification between predicted and known diets (nine for diet and 35 for locomotion) are labelled with the originally assigned category.

In our brief exploration of how the species in our sample occupy a morphospace with Euclidean properties, the nMDS with Sammon's nonlinear mapping allowed us to construct a new lower-dimensional dataset with a structure as similar to the original morphometric dataset as possible. We combined this two-dimensional space with the predictions of the discriminant analyses to visualize the overlap or separation of species with different ecological habits in morphological space. For diet types: generalized herbivores occupied a vast portion of the morphospace (figure 3*a*) without much overlap with the other three diet types. In terms of locomotor modes, terrestrial species overlapped considerably with arboreal, gliding and semifossorial species (figure 3*b*) but not with the other locomotor modes (ricochetal, semiaquatic and fossorial) that have marked structural requirements (e.g. elongated hind feet that increase stride length when jumping or robust appendages for digging).

## 4. Discussion

For the most part, rodents have a conserved and recognizable body plan. Overall, they are stout-bodied, short-limbed and long-tailed small mammals with a conserved suite of craniodental features for gnawing [57]. Nonetheless, rodents are the most successful group in the evolutionary history of mammals in terms of taxonomic diversity, and there is at once remarkable diversification and extreme conservatism of morphological characters. After controlling for differences in body size and quantifying the effects of phylogenetic covariance, we found that unrelated species sharing diet and/or locomotion types are characterized by shared morphological features, a likely indication of widespread homoplasy and convergent evolution.

### 4.1. Phylogenetic patterns

We found very low phylogenetic signals for our chosen morphological and ecological traits, and no difference in the results from phylogenetically informed and non-phylogenetic discriminant analyses. Despite this finding, we still consider that testing for phylogenetic signal and using a canonical variates analysis that can account for phylogenetic non-independence is a crucial first step in ecomorphological studies. Convergent evolution and parallelism in rodent feeding and locomotion has been documented [58], so a species might share the same ecological attributes (and the corresponding morphology) with its close relatives but also with distantly related species that radiated into a similar niche. Low phylogenetic signal is probably diminished further by the fact that, in rodents, species with different ecologies can have relatively similar morphologies [30]. Also, there can be multiple adaptive solutions to a selective regime [2]. For example, fossorial species can be either scratch-diggers or tooth-diggers (e.g. gophers and naked mole rats, respectively), and some have substantial morphological modifications suited to each digging mode [59]. Conversely, similar features can evolve in response to different adaptive regimes (e.g. both ricochetal and arboreal species have very long tails).

### 4.2. Ecological relevance of morphological traits

In ecomorphological studies, the question of how many and which characters to measure is crucial for the meaningful interpretation of morphological space. Our results support the *a priori* expectation for ecological relevance of the chosen variables. This correlative approach is no replacement for fine-scale studies that explicitly consider biomechanics and performance when examining the form–function relationship [60], but we consider that our whole-body, collections-based approach provides a useful base for interpreting species' morphologies at the order level. We used all craniodental and external characters together because they do not represent separate systems with unique biological properties, and their contribution to the discriminant analyses reflects common functional demands. Food resources depend on the way in which they are obtained, and most behaviour involves locomotion [61]. For example: two external characters showed high discriminant power in relation to diet type: ear length (E) and vibrissae length (Vib). These sensory traits play a role in finding food, foraging and prey capture [62]. We found that vibrissae were longer (in relation to body size) in arboreal species than they were in terrestrial, fossorial or semiaquatic species. This result supports the notion that vibrissae are important for orientation in low-visibility conditions and discontinuous substrates, such as tree branches at night time [63]. Craniodental characters proved useful in predicting locomotion type, perhaps because they are involved in posture and digging, and because the skull carries important sensory organs [64].

The angle of the condylar process (ACP) stood out as a powerful indicator of diet and locomotion strategy. This measurement is not commonly used in studies of rodent morphology, but it captured the biomechanical consequences of gape angle and bite force on the occlusal apparatus. Specialized herbivores with robust mandibles, wide gapes and strong bite forces have higher ACP values, while animal-eating species have narrow ACP angles with gracile mandibles and reduced dentition. Different values of ACP relate with other cranial and mandibular features (molars, jaw articulation, zygomatic structure), including subtle changes that influence the insertion of the masticatory part of the masseter, temporal and pterygoid muscles. Given the abundance of rodent mandibles in fossil record, ACP represents a new opportunity for morphological inference of the ecology of fossil rodents and subsequent analyses of the ecological dynamics of palaeocommunities.

### 4.3. Discrimination of ecological categories

We were interested in how morphology predicts ecology, and the ecological relevance of our chosen measurements. Assigning all the species in the training data into their ‘correct’ ecology was not our objective, and we consider most misclassification in our models as a result of the ecological versatility of rodents, multiple adaptive solutions to similar selective regimes, uncertainty in the ecological literature, and in the way we interpreted vague or conflicting verbal descriptions of feeding and substrate use into discrete classes. While our results suggest widespread convergence and parallelism in rodent ecology, we did not explicitly test the hypothesis of convergent evolution. Recent comparative methods [65,66] that can detect and measure convergent evolution can be readily applied to our data. This investigation of convergence represents a rewarding topic that would enrich our present findings, as long as the evolutionary models involved in these tests are applied and interpreted correctly [67].

Certain ecological strategies are worth discussing in light of our results, such as the differences between and within plant eaters and animal eaters. It is not biologically meaningful to separate generalized herbivores and omnivores. Despite widespread use in the recent literature, omnivory is not a cohesive category in the case of rodents. Generalized herbivores consume plant parts with little or no cellulose (fruit, seeds, gum and nectar) and opportunistically consume animal matter and fungi. Specialized herbivores separated from other diet classes in the nMDS morphospace (figure 3). They consume the vegetative parts of plants (leaves, stems and roots), which often contain grit and silica. These foods are procured and processed with a suite of cranial features suited for maximum bite force when nibbling and cutting tough, fibrous foods, while maximizing grinding pressure to break down plant matter before ingestion. The morphological ‘cluster’ of specialist herbivores included Arvicolines (voles and relatives) with varied lifestyles (semiaquatic, terrestrial and semifossorial), subterranean gophers and the European beaver, known for gnawing and grinding wood.

Specialized herbivores have exclusively vegetarian diets, because the symbiotic microorganisms in their digestive tracts that help them break down cellulose require a more or less constant environment. Some species which we classified as specialized herbivores were not predicted as such on the basis of morphology despite having well-documented herbivorous diets. These species occupied the morphospace between generalized and specialized herbivores, since they do not share the well-developed zygomasseteric complex of voles, gophers or beavers. The Australian broad-toothed mouse (*Mastacomys fuscus*) mostly eats grasses and is an example of such a specialized herbivore. Despite having broad teeth to clip pliable but tough grasses and a long maxillary tooth row to grind them, this species is morphologically closer to other generalized herbivores and is most probably meeting demands imposed by a herbivorous diet through a specialized gut morphology and with broader, more complex molars [68]. The Mexican hairy dwarf porcupine (*Sphiggurus mexicanus*) is another strict vegetarian, but its arboreal habits probably give it access to young leaves and leaf buds that do not require extensive craniodental modifications to chew.

The separation of species that specialize on soft-bodied invertebrates (insectivorous) from those that consume more varied animal matter (carnivorous, consuming chitinous invertebrates and vertebrates) is ecologically sound in light of our results. From limited ecological data, we originally classified both species of *Pseudohydromys* as insectivorous, but the discriminant analysis classified the eastern moss mouse (*P. murinus*) as carnivorous. These two species are on opposite ends of the spectrum of molar reduction within the New Guinea moss mice radiation, and it is likely that of the two, only *P. ellermani* specializes on soft-bodied invertebrates that need no chewing. We had originally assigned the white-tailed antelope squirrel (*Ammospermophilus leucurus*) as a generalized herbivore, but under our model it was classified as carnivorous on the basis of morphology. The predatory propensity of ground squirrels is well documented [69], and in this case these habits seem to be reflected in their craniodental morphology.

Gliding, fossorial and jumping forms present clear differences in appendicular measurements and were all correctly classified. Fossorial forms present the longest claws, jumping species have long tails and hind feet and shorter forefeet, and gliding squirrels have proportionally longer limbs than non-gliding forms. Our choice of morphological characters yielded good classification of semiaquatic species even if we did not consider known swimming features such as waterproof fur, webbed feet and flattened tails. Despite having different diets and using different parts of the aquatic medium, all the semiaquatic species except the Rakali (Australian water rat, *Hydromys chrysogaster*) were correctly classified by our model. Like other swimming forms, this large predatory rat has small ears and stout whiskers, but it does not share the limb proportions of the other swimming rodents in the dataset. Terrestrial forms overlap with both semifosorrial and arboreal forms in morphological space, leading to increased misclassification in these three categories. For example, many deer mice, melomys and harvest mice are excellent climbers with ‘all-purpose’ morphologies, of which only a few species have been documented to lead truly arboreal lifestyles. Similarly, the relationship between burrow use, digging ability and morphological modifications are still unclear.

## 5. Conclusion

This study provides a useful and accessible set of characters for ecomorphological analysis and adds to our understanding of mammalian form and function. Nonetheless, we must continue collecting morphological data and make efforts to synthesize the wealth of existing ecological data for such an important order of mammals. The species we analysed were restricted to four biogeographic realms, and several families with potentially unique morphological specializations are not represented. Species from a wider geographical scope should be analysed in further studies. Because of the weak relationship between phylogeny and ecomorphology, we cannot predict the ecological similarity between species adequately using only phylogenies. However, ecological metrics derived from morphology can overcome the limitations of limited ecological data and convergent evolution in comparative studies. Voucher specimens are valuable records of species' morphological phenotypes, and with the suitable set of characters used here, morphological similarity can be used to quantify functional similarity, an important component of research into community ecology, palaeoecology and macroecology. Our approach highlights the important role that museum collections can play in not just taxonomic or phylogenetic studies, but also in advancing the study of ecology [70,71].

## Supplementary Material

Appendix: contains data definitions for the files in the online repository, supplementary tables 1 and 2, supplementary figure 1, and additional references.

## Data accessibility

Data and R code are available as an archived GitHub repository [72], available at http://doi.org/10.5281/zenodo.201147.
