# Supplementary material for: Morphology captures diet and locomotor types in rodents
Source: R Soc Open Sci. 2017 Jan 18;4(1):160957. doi: 10.1098/rsos.160957 (PMC5319359; doi:10.1098/rsos.160957)
Supplement: Appendix: contains data definitions for the files in the online repository, supplementary tables 1 and 2, supplementary figure 1, and additional references. [file rsos160957supp1.pdf]

Royal Society Open Science

Supplementary material for:

Morphology captures diet and locomotor types in rodents

Authors: Luis D. Verde Arregoitia, Diana O. Fisher, and Manuel Schweizer

Data definition

The following files can be downloaded from:

http://doi.org/10.5281/zenodo.201147

Ecological data (DietLocomotion.csv). Diet type and locomotion categories and the data sources used to assign them, following the numbered reference lists below (Reference Lists 3 and 4).

Body size data (bodySizes.csv). Body size data for all 208 species with data sources, following the numbered reference list below (Reference List 2).

Specimens examined (measurementsFinal.csv). Original measurements taken by LDVA. The *mus* field identifies the collections that house the specimens. QM = Queensland Museum (Brisbane, Australia), AusMus = Australian Museum (Sydney, Australia), MZFC = ‘‘Alfonso L. Herrera’’ Zoology Museum (UNAM, Mexico City, Mexico). See main text for measurement methods and definitions.

Species means (meansMasses.csv). Mean values for all 14 measurements with their data source. Values derived from specimens measured by LDVA are identified with in the *dataProv* field as “LD”. Other values were collated from the sources in Reference List 1 and updated from amended tables and correspondence with the lead author (identified in the table as “AM”). Sources for body mass data are detailed separately. See main text for measurement methods and definitions.

All other tables are produced as intermediate or final outputs of the analysis scripts provided The R scripts are named in the order in which they can be used. “phylo.fda.v0.2noPlotting” needs to be sourced for several of the analyses.

Table S1. Taxonomic context of species examined in this study.

Family	Total species	Species examined
MURIDAE	719	54
CRICETIDAE	710	84
SCIURIDAE	279	7
ECHIMYIDAE	90	0
HETEROMYIDAE	62	14
NESOMYIDAE	61	15
CTENOMYIDAE	60	0
DIPODIDAE	50	28
GEOMYIDAE	39	1
GLIRIDAE	29	3
CAPROMYIDAE	27	0
SPALACIDAE	21	0
CAVIIDAE	19	0
ERETHIZONTIDAE	18	1
BATHYERGIDAE	15	0
DASYPROCTIDAE	13	0
HYSTRICIDAE	13	0
OCTODONTIDAE	13	0
ABROCOMIDAE	10	0
CALOMYSCIDAE	8	0
ANOMALURIDAE	7	0
CHINCHILLIDAE	7	0
CTENODACTYLIDAE	5	0
CASTORIDAE	3	1
CUNICULIDAE	2	0
PEDETIDAE	2	0
PLATACANTHOMYIDAE	2	0
THRYONOMYIDAE	2	0
APLODONTIIDAE	1	0
DIATOMYIDAE	1	0
DINOMYIDAE	1	0
MYOCASTORIDAE	1	0
PETROMURIDAE	1	0

Table S2. Parameter estimates from PGLS size correction (mean and 95% CI for 100 phylogenetic trees)

Character	intercept	intercept 95% CI	slope	slope 95% CI
LR	1.129	0.007	0.252	0.001
ZB	1.916	0.007	0.25	0.001
BIT	-0.564	0.004	0.307	0.001
LMT	0.475	0.007	0.299	0.001
HMC	0.432	0.006	0.3	0.001
T	3.495	0.01	0.292	0.002
E	1.985	0.01	0.188	0.002
Vib	2.699	0.016	0.237	0.003
HF	2.232	0.011	0.29	0.002
FF	1.315	0.009	0.291	0.002
UM	-0.701	0.011	0.323	0.002

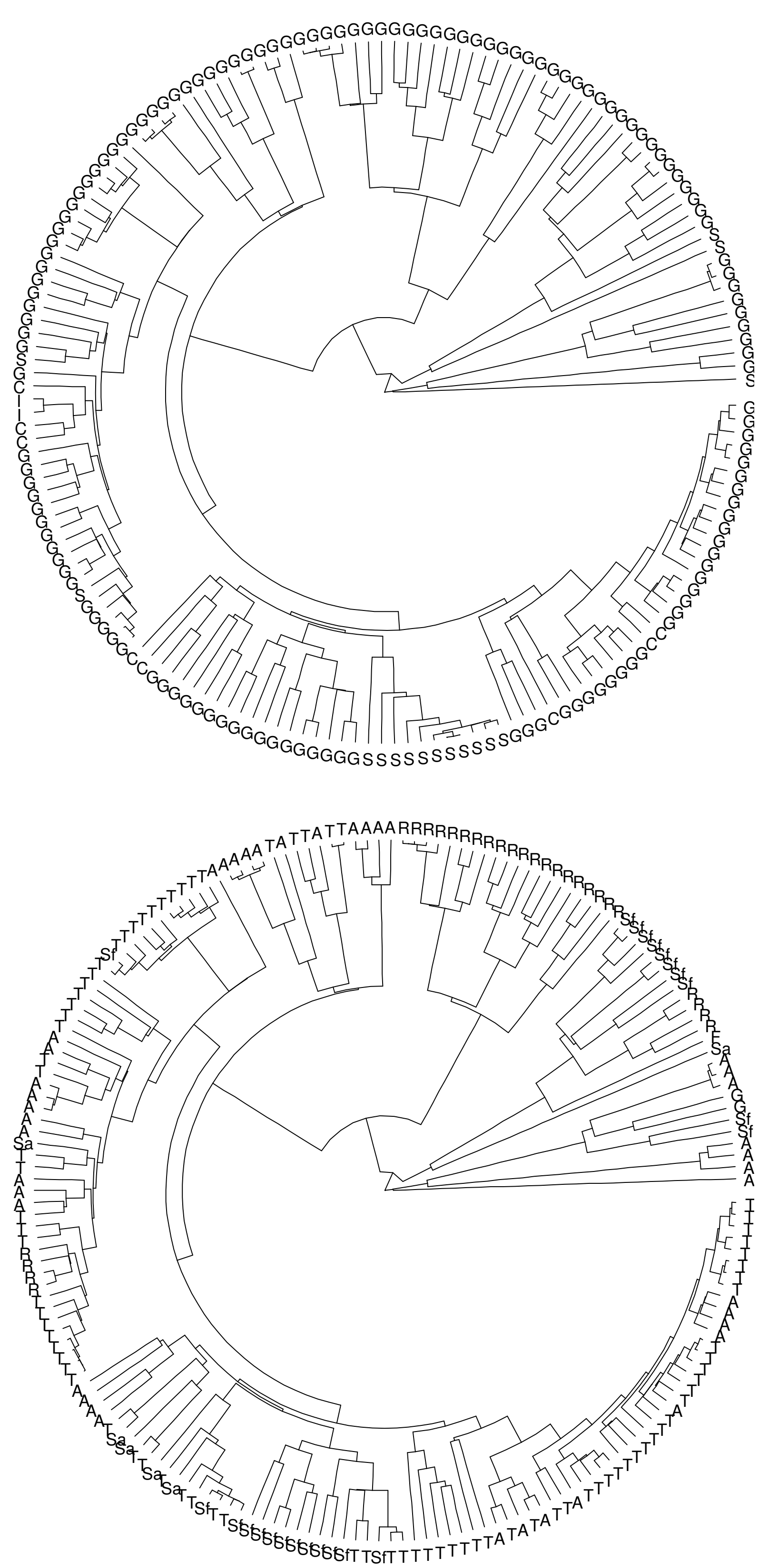

Figure S1. Distribution of diet types and locomotor modes on a randomly-selected phylogenetic tree (from the set of 100) from the mammalian phylogeny of Faurby and Svenning (2015). Abbreviatons for diet types - C: carnivore, G: generalized herbivore, I: insectivore, S: specialized herbivore. Abbreviatons for locomotion modes - T: terrestrial, Sa: semiaquatic A: arboreal, Sf: semifossorial, F: fossorial, R: ricochetal, G: gliding.

Reference List 1.

Miljutin, A. (1997) *Ecomorphology of the Baltic Rodents: Body Form, Ecological Strategies, and Adaptive Evolution*. Teaduste Akadeemia Kirjastus.

Miljutin, A. (1998) Ecological Strategies of the Baltic Rodents. *Proceedings of the Latvian Academy of Sciences. Section B,* **52,** 20-30.

Miljutin, A. (1999) Trends of specialization in rodents: the birch mice, genus *Sicista* (Dipodoidea, Rodentia). *Folia Theriologica Estonica,* **4,** 76-90.

Miljutin, A. (2006) African climbing mice (Dendromus, Muroidea) and Palaearctic birch mice (Sicista, Dipodoidea): an example of parallel evolution Among Rodents. *Acta Zoologica Lituanica,* **16,** 84-92.

Miljutin, A. (2008) Trends of Specialisation in Rodents: The Five-Toed Jerboas, Subfamily Allactaginae (Dipodoidea, Rodentia). *Acta Zoologica Lituanica,* **18,** 228-239.

Miljutin, A. (2011) Trends of Specialisation in Rodents: The Hamsters, Subfamily Cricetinae (Cricetidae, Rodentia, Mammalia). *Acta Zoologica Lituanica,* **21,** 192-206.

Miljutin, A. & Lehtonen, J.T. (2008) Probability of competition between introduced and native rodents in Madagascar: An estimation based on morphological traits. *Estonian Journal of Ecology,* **57,** 133-152.

Reference List 2. Body size data sources

1. Specimen label data collected by LDVA
2. Specimen label data from the work of Andrei Miljutin
3. Coe, M.J. & Foster, J.B. (1972) The mammals of the northern slopes of Mt Kenya *Journal of the East African Natural History Society Museum,* **131,** 1-18.
4. Jones, K.E., Bielby, J., Cardillo, M., Fritz, S.A., O'Dell, J., Orme, C.D.L., Safi, K., Sechrest, W., Boakes, E.H., Carbone, C., Connolly, C., Cutts, M.J., Foster, J.K., Grenyer, R., Habib, M., Plaster, C.A., Price, S.A., Rigby, E.A., Rist, J., Teacher, A., Bininda-Emonds, O.R.P., Gittleman, J.L., Mace, G.M., Purvis, A. & Michener, W.K. (2009) PanTHERIA: a species-level database of life history, ecology, and geography of extant and recently extinct mammals. *Ecology,* **90,** 2648-2648.
5. Kays, R.W. & Wilson, D.E. (2009) *Mammals of North America,* Second Edition. Princeton University Press, Princeton, New Jersey.
6. Lawes, M.J., Fisher, D.O., Johnson, C.N., Blomberg, S.P., Frank, A.S.K., Fritz, S.A., McCallum, H., VanDerWal, J., Abbott, B.N., Legge, S., Letnic, M., Thomas, C.R., Thurgate, N., Fisher, A., Gordon, I.J. & Kutt, A. (2015) Correlates of Recent Declines of Rodents in Northern and Southern Australia: Habitat Structure Is Critical. *PLoS ONE,* **10,** e0130626.
7. Marquart, K. (2015) Habitat use and morphological adaptations of endemic rodents (Muroidea: Nesomyinae) of East Madagascar. PhD Thesis, Universität Hohenheim.
8. Smith, A.T., Xie, Y., Hoffmann, R.S., Lunde, D., MacKinnon, J., Wilson, D.E. & Wozencraft, W.C. (2010) *A guide to the mammals of China.* Princeton University Press.

Reference List 3. Diet data sources

1. Abt, K. & Bock, W. (1998) Seasonal variations of diet composition in farmland field mice Apodemus spp. and bank voles Clethrionomys glareolus. *Acta Theriologica,* **43,** 379-389.
2. Álvarez-Castañeda, S.T. (2005) Peromyscus melanotis. *Mammalian Species,* **764,** 1-4.
3. Alvarez, T. & Mayo-Aceves, E. (1993) *Contribución al conocimiento de los hábitos alimentarios del ratón de los volcanes, Neotomodon alstoni,(Merriam, 1898).* Instituto de Ecología.
4. Best, T.L. (1994) Chaetodipus nelsoni. *Mammalian Species,* **484,** 1-6.
5. Best, T.L. & Skupski, M.P. (1994a) Perognathus flavus. *Mammalian Species,* **471,** 1-10.
6. Best, T.L. & Skupski, M.P. (1994b) Perognathus merriami. *Mammalian Species,* **473,** 1-7.
7. Ceballos, G. (2005) Habromys ixtlani. *Los Mamíferos Silvestres de México* (eds G. Ceballos & G. Oliva), pp. 672. Fondo de Cultura Económica-CONABIO, Mexico D.F.
8. Ceballos, G. (2014) *Mammals of Mexico*. Johns Hopkins University Press.
9. Colak, E. & Yigit, N. (1998) A new subspecies of Jerboa from Turkey: Allactaga euphratica kivanci* subsp. n. *Turkish Journal of Zoology,* **22,** 93-98.
10. Dammhahn, M., Soarimalala, V. & Goodman, S.M. (2013) Trophic Niche Differentiation and Microhabitat Utilization in a Species-rich Montane Forest Small Mammal Community of Eastern Madagascar. *Biotropica,* **45,** 111-118.
11. Eshelman, B.D. & Cameron, G.N. (1987) Baiomys taylori. *Mammalian Species Archive,* **285,** 1-7.
12. González-Espinosa, M. & Quintana-Ascencio, P.F. (1986) Seed predation and dispersal in a dominant desert plant: Opuntia, ants, birds, and mammals. *Frugivores and seed dispersal* (eds A. Estrada & T.H. Fleming), pp. 273-284. Springer Netherlands, Dordrecht.
13. González-Ruiz, N., Ramírez-Pulido, J. & Genoways, H.H. (2009) Review of the harvest mice (Genus reithrodontomys) in the Mexican state of Mexico. *Western North American Naturalist,* **67,** 238-250.
14. González-Salazar, C., Martínez-Meyer, E. & López-Santiago, G. (2014) A hierarchical classification of trophic guilds for North American birds and mammals. *Revista Mexicana de Biodiversidad,* **85,** 931-941.
15. Helgen, K.M. & Helgen, L.E. (2009) Chapter 8. Biodiversity and Biogeography of the Moss-mice of New Guinea: A Taxonomic Revision of Pseudohydromys (Muridae: Murinae). *Bulletin of the American Museum of Natural History***,** 230-313.
16. Henderson, C.B. (1990) The Influence of Seed Apparency, Nutrient Content and Chemical Defenses on Dietary Preference in Dipodomys ordii. *Oecologia,* **82,** 333-341.
17. Hernández-Betancourt, S., Gómez González, J., Cimé Pool, J., Medina Peralta, S. & Canul, E. (2005) First report of use of land snails for Heteromys gaumeri (Rodentia: Heteromyidae) in a subdeciduous forest in Yucatán, México. *Acta Zoologica Mexicana,* **21,** 155-156.
18. Hooper, E.T. (1968) Habitats and Food of Amphibious Mice of the Genus Rheomys. *Journal of Mammalogy,* **49,** 550-553.
19. Hunt, J.L., Morris, J.E. & Best, T.L. (2004) *Nyctomys sumichrasti. Mammalian Species,* **754,** 1-6.

20. Jack, I. & Rasoazanabary, E. (2008) Discovery of Macrotarsomys bastardi at Beza Mahafaly Special Reserve, southwest Madagascar, with observations on the dynamics of small mammal interactions. *Madagascar Conservation & Development,* **3**.
21. Kelt, D.A., Brown, J.H., Heske, E.J., Marquet, P.A., Morton, S.R., Julian, R.W.R., rogovin, K., xed, n, A. & Shenbrot, G. (1996) Community Structure of Desert Small Mammals: Comparisons Across Four Continents. *Ecology,* **77,** 746-761.
22. Kissling, W.D., Dalby, L., Fløjgaard, C., Lenoir, J., Sandel, B., Sandom, C., Trøjelsgaard, K. & Svenning, J.-C. (2014) Establishing macroecological trait datasets: digitalization, extrapolation, and validation of diet preferences in terrestrial mammals worldwide. *Ecology and Evolution,* **4,** 2913-2930.
23. Lacey, E.A. (2000) *Life Underground: The Biology of Subterranean Rodents.* University of Chicago Press, USA.
24. López-Wilchis, R. & Torres-Flores, J.W. (2007) Diet of the Jalapan Pine Vole (Microtus quasiater) in Mature Mountain Cloud Forest. *Journal of Mammalogy,* **88,** 515-518.
25. Macswiney G., M.C., Hernandez-Betancourt, S. & Avila-Flores, R. (2009) *Otonyctomys hatti. Mammalian Species,* **825,** 1-5.
26. Martin, S.A. (2010) Dental Adaptation in Murine Rodents (Muridae): Assessing Mechanical Predictions. M.Sc. Thesis, Florida State University.
27. Miljutin, A. & Lehtonen, J.T. (2008) Probability of competition between introduced and native rodents in Madagascar: An estimation based on morphological traits. *Estonian Journal of Ecology,* **57,** 133-152.
28. Mulungu, L.S., Themb'alilahlwa, A.M., Massawe, A.W., Kennis, J., Crauwels, D., Eiseb, S., Monadjem, A., Makundi, R.H., Katakweba, A.A. & Leirs, H. (2011) Dietary differences of the multimammate mouse, Mastomys natalensis (Smith, 1834), across different habitats and seasons in Tanzania and Swaziland. *Wildlife Research,* **38,** 640-646.
29. Nowak, R.M. (1999) *Walker's mammals of the world.* John Hopkins University, Baltimore, Maryland.
30. Oaks, E.C., Young, P.J., Kirland, G.L. & Schimdt, D.F. (1987) Spermophilus variegatus. *Mammalian Species,* **272,** 1-8.
31. Packard, R.L. & Montgomery, J.B. (1978) Baiomys musculus. *Mammalian Species*, 1-3.
32. Preston, S.D. & Jacobs, L.F. (2005) Cache decision making: the effects of competition on cache decisions in Merriam's kangaroo rat (Dipodomys merriami). *Journal of Comparative Psychology,* **119,** 187.
33. Price, S.A. & Hopkins, S.S.B. (2015) The macroevolutionary relationship between diet and body mass across mammals. *Biological Journal of the Linnean Society,* **115,** 173-184.
34. Reid, F. (2006) *A field guide to mammals of North America, north of Mexico.* Houghton Mifflin Harcourt.
35. Rickart, E.A. & Robertson, P.B. (1985) Peromyscus melanocarpus. *Mammalian Species*, 1-3.
36. Rowe, K.C., Achmadi, A.S. & Esselstyn, J.A. (2016) Repeated evolution of carnivory among Indo-Australian rodents. *Evolution,* **70,** 653-665.
37. Rowe, K.C., Reno, M.L., Richmond, D.M., Adkins, R.M. & Steppan, S.J. (2008) Pliocene colonization and adaptive radiations in Australia and New Guinea (Sahul): Multilocus systematics of the old endemic rodents (Muroidea: Murinae). *Molecular Phylogenetics and Evolution,* **47,** 84-101.

38. Ryan, J.M. (2003) Nesomys, Red Forest Rat, Voalavo Mena. *The Natural History of Madagascar* (eds S.M. Goodman & J.P. Benstead). The University of Chicago Press, Chicago, USA and London, UK.
39. Samuels, J.X. (2009) Cranial morphology and dietary habits of rodents. *Zoological Journal of the Linnean Society,* **156,** 864-888.
40. Sánchez-Hernández, C., Schnell, G.D. & Romero-Almaraz, M.d.L. (2009) Peromyscus perfulvus (Rodentia: Cricetidae). *Mammalian Species***,** 1-8.
41. Shenbrot, G.I., Krasnov, B. & Rogovin, K.A. (2012) *Spatial Ecology of Desert Rodent Communities*. Springer Berlin Heidelberg.
42. Smith, A.T., Xie, Y., Hoffmann, R.S., Lunde, D., MacKinnon, J., Wilson, D.E. & Wozencraft, W.C. (2010) *A guide to the mammals of China*. Princeton University Press.
43. Tiphaine, C., Yaowalak, C., Cyril, C., Helder, G.-R., Jacques, M., Paul, T., Monique, V.-L., Laurent, V. & Vincent, L. (2013) Correlated changes in occlusal pattern and diet in stem Murinae during the onset of the radioation of old world rats and mice *Evolution,* **67,** 3323-3338.
44. Vázquez, L.B., Cameron, G.N. & Medellín, R.A. (2001) Peromyscus aztecus. *Mammalian Species,* **649,** 1-4.
45. Vázquez, L.B., Cameron, G.N. & Medellín, R.A. (2004) Characteristics of Diet of Peromyscus aztecus and Reithrodontomys fulvescens in Montane Western Mexico. *Journal of Mammalogy,* **85,** 196-205.
46. Yiğit, N., Kankiliç, T. & Çolak, E. (2007) Reproductive biology and postnatal development of Microtus rossiaemeridionalis Ognev, 1924 (Mammalia: Rodentia) distributed in Turkey. *Turkish Journal of Zoology,* **31,** 287-294.

Reference List 4. Locomotion data sources

1. Álvarez-Castañeda, S.T. (2005) Peromyscus melanotis. *Mammalian Species,* **764,** 1-4.
2. Angelici, F.M. & Luiselli, L. (2005) Patterns of specific diversity and population size in small mammals from arboreal and ground-dwelling guilds of a forest area in southern Nigeria. *Journal of Zoology,* **265,** 9-16.
3. Bartholomew, G.A. & Caswell, H.H. (1951) Locomotion in Kangaroo Rats and Its Adaptive Significance. *Journal of Mammalogy,* **32,** 155-169.
4. Berry, A., Anderson, T., Amos, J. & Cook, J. (1987) Spool-and-line tracking of giant rats in New Guinea. *Journal of Zoology,* **213,** 299-303.
5. Best, T.L. & Skupski, M.P. (1994a) Perognathus flavus. *Mammalian Species,* **471,** 1-10.
6. Best, T.L. & Skupski, M.P. (1994b) Perognathus merriami. *Mammalian Species,* **473,** 1-7.
7. Buesching, C.D., Newman, C., Twell, R. & Macdonald, D.W. (2008) Reasons for arboreality in wood mice *Apodemus sylvaticus* and Bank voles *Myodes glareolus*. *Mammalian Biology - Zeitschrift fur Saugetierkunde,* **73,** 318-324.
8. Castañeda-Rico, S., León-Paniagua, L., Ruedas, L.A. & Vázquez-Domínguez, E. (2011) High genetic diversity and extreme differentiation in the two remaining populations of Habromys simulatus. *Journal of Mammalogy,* **92,** 963-973.
9. Ceballos, G. (2014) *Mammals of Mexico*. Johns Hopkins University Press.
10. Djawdan, M. & Garland, T. (1988) Maximal running speeds of bipedal and quadrupedal rodents. *Journal of Mammalogy,* **69,** 765-772.

11. Dobly, A. & Rozenfeld, F.M. (2000) Burrowing by common voles (Microtus arvalis) in various social environments. *Behaviour,* **137,** 1443-1461.
12. Dwyer, P. (1978) A Study of *Rattus exulans* (Peale) (Rodentia : Muridae) in the New Guinea Highlands. *Wildlife Research,* **5,** 221-248.
13. Dwyer, P.D. (1982) Prey switching: A case study from New Guinea. *The Journal of Animal Ecology*, 529-542.
14. Etheredge, D.R., Engstrom, M.D. & Stone, R.C. (1989) Habitat Discrimination between Sympatric Populations of Peromyscus attwateri and Peromyscus pectoralis in West-Central Texas. *Journal of Mammalogy,* **70,** 300-307.
15. Feldhamer, G.A., Thompson, B.C. & Chapman, J.A. (2003) *Wild mammals of North America: biology, management, and conservation*. JHU Press.
16. Flannery, T. & Seri, L. (1990) The mammals of southern West Sepik Province, Papua New Guinea: their distribution, abundance, human use and zoogeography. *Records of the Australian Museum,* **42,** 173-208.
17. Flannery, T.F. (1995) *Mammals of new guinea*. Reed.
18. Francis, C.M. & Barrett, P. (2008) *A Field Guide to the Mammals of South-East Asia*. New Holland.
19. García Mendoza, D.F. & López González, C. (2005) Diminutive woodrat (Nelsonia neotomodon) in Chihuahua, Mexico. *The Southwestern Naturalist,* **50,** 503-506.
20. González-Salazar, C., Martínez-Meyer, E. & López-Santiago, G. (2014) A hierarchical classification of trophic guilds for North American birds and mammals. *Revista Mexicana de Biodiversidad,* **85,** 931-941.
21. Haffner, M. (1998) A comparison of the gross morphology and micro-anatomy of the foot pads in two fossorial and two climbing rodents (Mammalia). *Journal of Zoology,* **244,** 287-294.
22. Hernández-Betancourt, S., Gómez González, J., Cimé Pool, J., Medina Peralta, S. & Canul, E. (2005) First report of use of land snails for Heteromys gaumeri (Rodentia: Heteromyidae) in a subdeciduous forest in Yucatán, México. *Acta Zoologica Mexicana,* **21,** 155-156.
23. Hooper, E.T. (1968) Habitats and Food of Amphibious Mice of the Genus Rheomys. *Journal of Mammalogy,* **49,** 550-553.
24. Hopkins, S.S.B. & Davis, E.B. (2009) Quantitative Morphological Proxies for Fossoriality in Small Mammals. *Journal of Mammalogy,* **90,** 1449-1460.
25. Hrachovy, S.K., Bradley, R.D. & Jones, C. (1996) Neotoma goldmani. *Mammalian Species,* **545,** 1-3.
26. Hunt, J.L., Morris, J.E. & Best, T.L. (2004) *Nyctomys sumichrasti. Mammalian Species,* **754,** 1-6.
27. IUCN (2013) IUCN Red List of Threatened Species. Version 2013.1.
28. Jekanoski, R.D. & Kaufman, D.W. (1995) Use of Simulated Herbaceous Canopy by Foraging Rodents. *American Midland Naturalist,* **133,** 304-311.
29. Kuncová, P. & Frynta, D. (2009) Interspecific morphometric variation in the postcranial skeleton in the genus Apodemus. *Belg. J. Zool,* **139,** 133-146.
30. León-Paniagua, L., Navarro-Sigüenza, A.G., Hernández-Baños, B.E. & Morales, J.C. (2007) Diversification of the arboreal mice of the genus Habromys (Rodentia: Cricetidae: Neotominae) in the Mesoamerican highlands. *Molecular Phylogenetics and Evolution,* **42,** 653-664.
31. Macswiney G., M.C., Hernandez-Betancourt, S. & Avila-Flores, R. (2009) *Otonyctomys hatti. Mammalian Species,* **825,** 1-5.

32. Miljutin, A. (2008) Trends of Specialisation in Rodents: The Five-Toed Jerboas, Subfamily Allactaginae (Dipodoidea, Rodentia). *Acta Zoologica Lituanica,* **18,** 228-239.
33. Miljutin, A. (2011) Trends of Specialisation in Rodents: The Hamsters, Subfamily Cricetinae (Cricetidae, Rodentia, Mammalia). *Acta Zoologica Lituanica,* **21,** 192-206.
34. Miljutin, A. & Lehtonen, J.T. (2008) Probability of competition between introduced and native rodents in Madagascar: An estimation based on morphological traits. *Estonian Journal of Ecology,* **57,** 133-152.
35. Musser, G.G., Helgen, K.M. & Lunde, D.P. (2008) Systematic review of New Guinea Leptomys (Muridae, Murinae) with descriptions of two new species. *American Museum Novitates***,** 1-60.
36. Nowak, R.M. (1999) *Walker's mammals of the world.* John Hopkins University, Baltimore, Maryland.
37. Oaks, E.C., Young, P.J., Kirland, G.L. & Schimdt, D.F. (1987) Spermophilus variegatus. *Mammalian Species,* **272,** 1-8.
38. Packard, R.L. & Montgomery, J.B. (1978) Baiomys musculus. *Mammalian Species***,** 1-3.
39. Reid, F.A. (2009) *A Field Guide to the Mammals of Central America and Southeast Mexico* Second edn. Oxford University Press, New York, NY.
40. Rickart, E.A. & Robertson, P.B. (1985) Peromyscus melanocarpus. *Mammalian Species***,** 1-3.
41. Ryan, J.M. (2003) Nesomys, Red Forest Rat, Voalavo Mena. *The Natural History of Madagascar* (eds S.M. Goodman & J.P. Benstead). The University of Chicago Press, Chicago, USA and London, UK.
42. Ryan, J.M., Creighton, G.K. & Emmons, L.H. (1993) Activity patterns of two species of Nesomys (Muridae: Nesomyinae) in a Madagascar rain forest. *Journal of Tropical Ecology,* **9,** 101-107.
43. Samuels, J.X. & Van Valkenburgh, B. (2008) Skeletal indicators of locomotor adaptations in living and extinct rodents. *Journal of Morphology,* **269,** 1387-1411.
44. Sánchez-Hernández, C., Schnell, G.D. & Romero-Almaraz, M.d.L. (2009) Peromyscus perfulvus (Rodentia: Cricetidae). *Mammalian Species***,** 1-8.
45. Shenbrot, G.I., Krasnov, B. & Rogovin, K.A. (2012) *Spatial Ecology of Desert Rodent Communities*. Springer Berlin Heidelberg.
46. Simeonovska-Nikolova, D. & Dekov, O. (2013) Some Aspects of the Behavior and Defensive Vocalization of the Romanian Hamster, Mesocricetus newtoni. *Acta Zoologica Bulgarica,* **65,** 461-468.
47. Smith, A.T., Xie, Y., Hoffmann, R.S., Lunde, D., MacKinnon, J., Wilson, D.E. & Wozencraft, W.C. (2010) *A guide to the mammals of China.* Princeton University Press.
48. Stein, B.R. (1989) Bone density and adaptation in semiaquatic mammals. *Journal of Mammalogy,* **70,** 467-476.
49. Stuart, C. & Stuart, T. (2001) *Field Guide to Mammals of Southern Africa.* Struik Publishers.
50. Van Dyck, S. & Strahan, R. (2008) *The mammals of Australia,* Third Edition. New Holland Publishers, Sydney.
51. Yiğit, N., Kankiliç, T. & Çolak, E. (2007) Reproductive biology and postnatal development of Microtus rossiaemeridionalis Ognev, 1924 (Mammalia: Rodentia) distributed in Turkey. *Turkish Journal of Zoology,* **31,** 287-294.
